# Role of ^18^F-choline PET/CT in evaluation of patients with prostate carcinoma

**DOI:** 10.2478/v10019-010-0050-8

**Published:** 2010-11-25

**Authors:** Marina Hodolic

**Affiliations:** Department for Nuclear Medicine, University Medical Centre Ljubljana, Slovenia

**Keywords:** prostate carcinoma, ^18^F-choline PET/CT, diagnosis, staging, follow-up

## Abstract

**Background:**

Choline presents a high affinity for malignant prostate tissue. It can be labelled with positron emitting ^18^F, and used for the evaluation of patients with prostate carcinoma by PET/CT imaging. The aim of this paper is to summarise our experience with fluoromethylcholine (^18^F-choline) PET/CT in patients with prostate cancer.

**Methods:**

In 4 months we investigated the patients with histopathological (or cytological) confirmed prostate cancer. Two observers evaluated the early and late ^18^F-choline PET images in correlation with corresponding localising CT images and using the semiquantitative standard uptake value (SUV) calculation.

**Results:**

The ^18^F-choline PET/CT was made in 50 patients with prostate cancer. There were 18 patients after radical prostatectomy and 32 without surgery. In all patients without surgery the pathological uptake was seen in the prostate. In 14 (44 %) patients of this group there was evidence of metastatic spread in local or distant lymph nodes and/or bones. In out of 18 patients after radical prostatectomy the local recurrence was detected in 6 patients (33%) and distant metastases were present in 2 patients (10%).

**Conclusions:**

^18^F-choline PET/CT seems to be useful imaging modality in patients with prostate carcinoma; it can demonstrate spread of the disease preoperatively and detect the local recurrence after radical prostatectomy.

## Introduction

Prostate carcinoma is the most common life-threatening cancer affecting men in the Western world. The mortality is around 10%. The major goals of pretherapeutic imaging are to determine the local extent of prostate carcinoma in terms of intraprostate localisation, extracapsular extension, seminal vesicle invasion, tumour infiltration into neurovascular bundles, surrounding tissues and organs in the small pelvis, detection of loco-regional metastases via the lymph nodes and detection of distant metastases. The exact pretherapeutic diagnosis and staging are essential, because the tumour treatment must be selected in strict dependence on the clinical tumour stage and risk profile.^1^,^2^

Both anatomic and functional molecular imaging of prostate carcinoma is important especially when there are problems with diagnosis, for example when prostate punch biopsies are negative while the suspicion of prostate carcinoma persists (for example rising PSA). They may also be helpful in localising the carcinoma, revealing how the carcinoma relates to the surrounding intra- and extraprostatic structures and organs.

^18^F-Fluorodeoxyglucose (FDG) PET/CT is a nuclear medicine procedure currently most widely used to diagnose primary and metastatic cancers.^3^ Unfortunately, not all tumours show a significant increase of metabolic activity on ^18^F-FDG PET/CT imaging. This is particularly true for neuroendocrine tumours, hepatic tumours and prostate cancer.^4^

Choline presents a high affinity for malignant prostate tissue, even if low grade. Choline can be labelled with either ^11^C or ^18^F, the former being the preference due to the lower urinary excretion and patients’ exposure. The latter is more useful for a possible distribution to centres lacking in on-site cyclotron. The sensitivity of ^18^F-choline PET/CT to detect prostate cancer preoperatively is 73%, greater than with ^18^F-FDG PET/CT (31%). Also the accuracy is greater with ^18^F-choline PET/CT (67%) than using ^18^F-FDG PET/CT (53%).^5^ The use of ^18^F-FDG in prostate cancer is limited to the most aggressive cancers.^6^

The aim of this paper is to summarise our experience with fluoromethylcholine (^18^F-choline) and PET/CT in patients with prostate cancer.

## Patients and methods

From 12.05.2010 to 15.09.2010 months we investigated the patients with cytological or histological confirmed prostate cancer.

The patients were fasting 6–10 hours prior the scan. ^18^F-choline (IASOcholine^®^ from IASON Austria) was injected *i.v.* (200 – 300 MBq, according to the weight of the patient) using the automatic radionuclide injector (Medrad). List mode acquisition over prostatic bed started immediately after the injection of the tracer and lasts for 5 minutes. After this early phase patients rested for approximately one hour. The whole body acquisition was performed thereafter, 2 min per bed position from base of the skull to midthigh (9 bed positions on average). Siemens Biograph mCT PET/CT scanner has been used.

**FIGURE 1.**
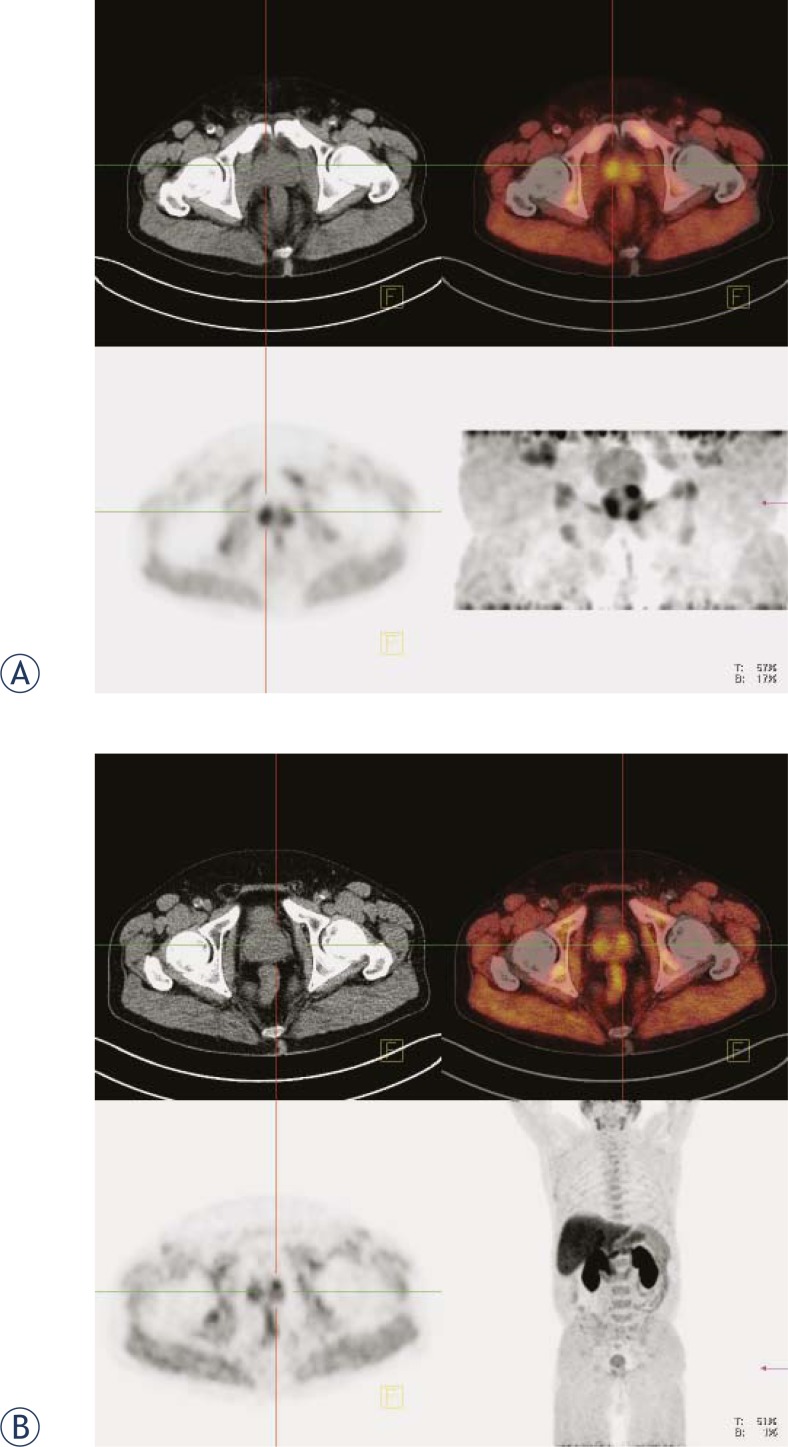
Prostate carcinoma: A prostatic bed (early images), B whole body (late images). Upper left panel: CT image. Upper right panel: fused PET/CT image. Lower left panel: PET image. Lower right panel: maximum intensity projection (MIP).

Early images were reconstructed from the list mode acquisition study before the activity appeared in the bladder (Figure 1A). Early (0–5 min *p.i*.) images and late (60 min *p.i*.) whole body images were presented in the usual transaxial, coronal and sagital planes. Two observers evaluated the images in correlation with corresponding localising CT images and using semiquantitative standard uptake value (SUV) calculation.

## Results

The ^18^F-choline PET-CT was performed in 50 patients with prostate cancer. The mean age was 67.7 years. There were 32 patients before radical prostatectomy and 18 after surgery (Table 1.).

**FIGURE 2.**
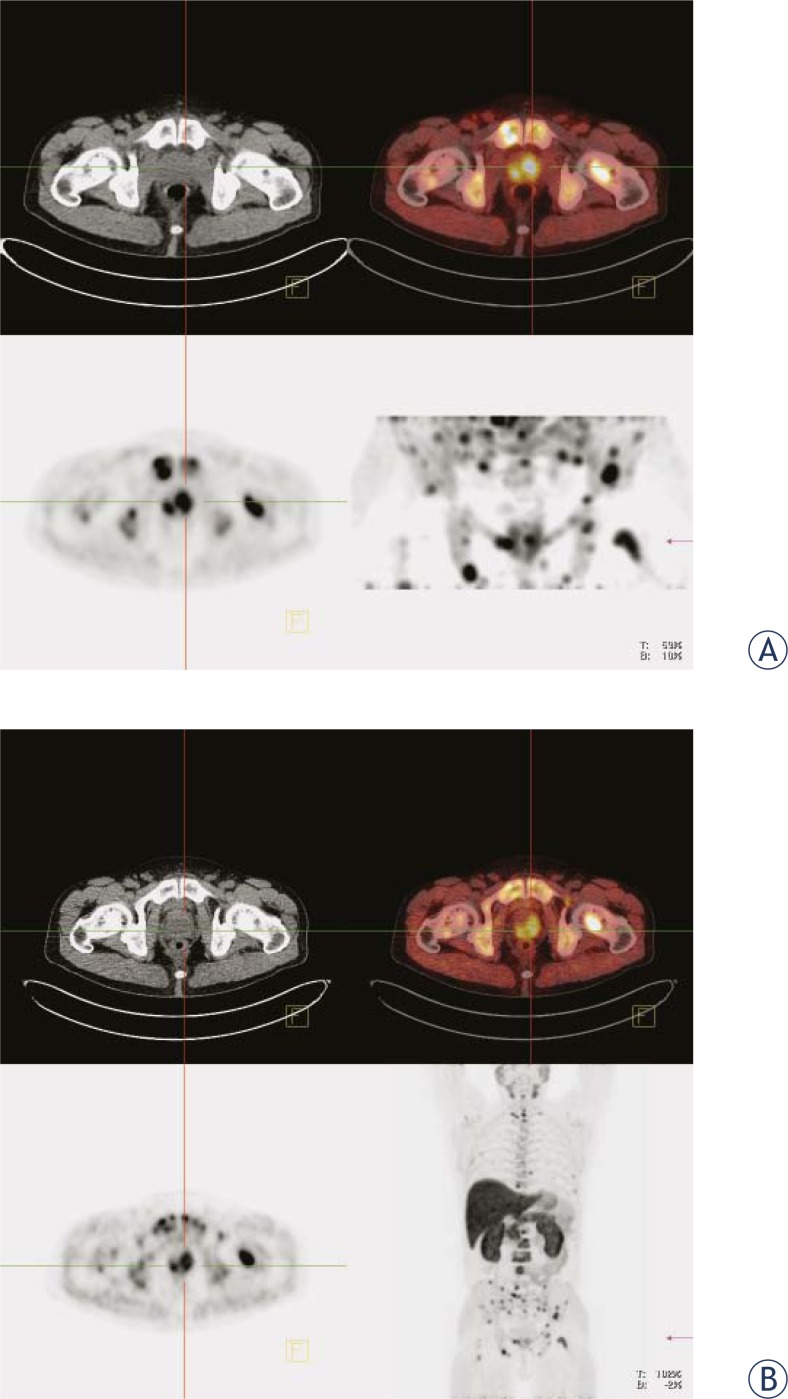
Bone metastases due to prostate cancer: A prostatic bed (early images), B whole body (late images). Upper left panel: CT image. Upper right panel: fused PET/CT image. Lower left panel: PET image. Lower right panel: maximum intensity projection (MIP).

The early phase has been used to evaluate prostate or prostate bed. The findings corresponded to late phase images in all patients (Figures 1 A, B). In patients with bony metastases in the pelvis the pathological uptake was seen in metastases already during the first 5 min after the tracer injection (Figures 2 A, B).

**FIGURE 3.**
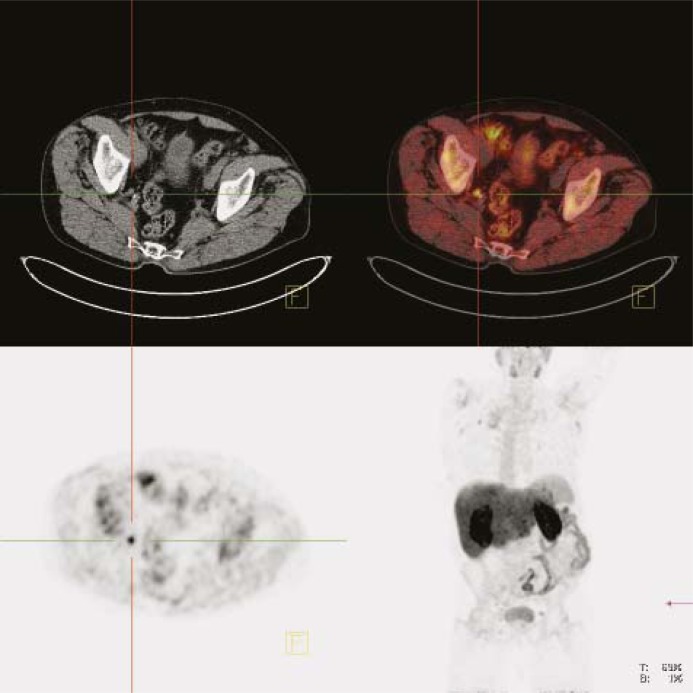
Lymph node metastases due to prostate cancer: whole body scan (late images). Upper left panel: CT image. Upper right panel: fused PET/CT image. Lower left panel: PET image. Lower right panel: maximum intensity projection (MIP).

**FIGURE 4.**
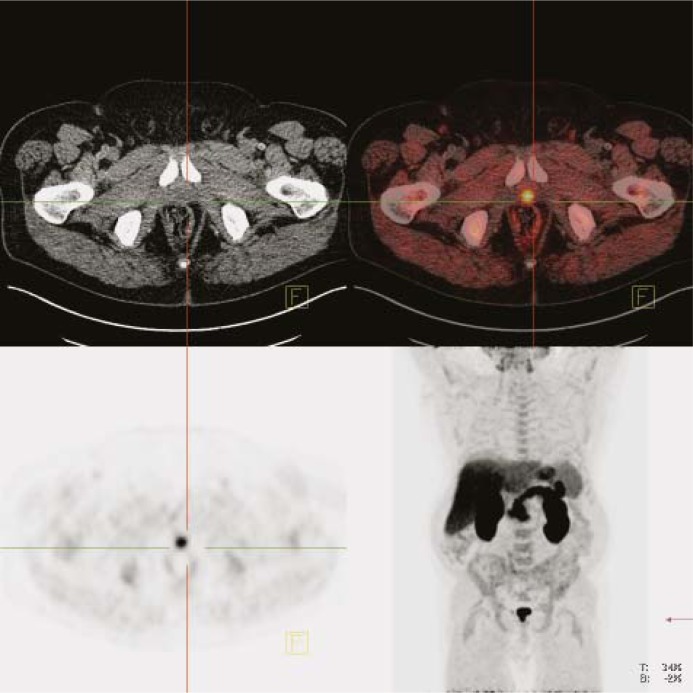
Relapse of prostate cancer: whole body (late images). Upper left panel: CT image. Upper right panel: fused PET/CT image. Lower left panel: PET image. Lower right panel: maximum intensity projection (MIP).

In all patients without surgery the pathological uptake was seen in the prostate. In 14 (44 %) patients of this group there was evidence of metastatic spread in local or distant lymph nodes (Figure 3) and/or bones (Figure 2 B). In patients after radical prostatectomy the local recurrence was detected in 6 patients (Figure 4) (33%) and distant metastases were present in 2 patients one had also the local recurrence; the other one has no evidence of local recurrent disease (Table 1).

## Discussion

Indications for ^18^F-choline PET/CT imaging modality in evaluation of patients with prostate cancer cover a wide spectrum of clinical settings: localisation of intraprostatic neoplastic lesions, initial staging, detection of occult recurrences and characterisation of images on conventional imaging modalities, which are questionable or difficult to interpret. ^18^F-choline is taken up by prostatic carcinoma as well as distant metastases very fast, already during 5 min after the injection (Figure 2).

The accurate knowledge of the normal biodistribution of ^18^F-choline is essential for the correct interpretation of PET/CT images. CT enables the differentiation of physiological bowel activity and ^18^F-choline excretion in the ureters. ^18^F-choline uptake in benign pathological conditions mainly includes sites of inflammation; nevertheless, the accumulation in tumour deposits not due to prostate cancer cannot be excluded.^7^

Similarly to FDG, choline is also taken up by infection.^8^ The differentiation between inflamed and metastatic lymph nodes can be achieved either by two phases PET or by appropriate antimicrobial treatment preceding ^18^F-choline PET/CT. On dual-phase PET of the prostate, areas of malignancy consistently demonstrated the stable or increasing ^18^F-choline uptake, whereas most areas containing benign tissue demonstrated the decreasing uptake.

Delayed or dual-phase imaging after the injection of ^18^F-choline may improve the performance of ^18^F-choline PET for localising malignant areas of the prostate.^9^ ^18^F-choline PET/CT showed a fast progressively increasing max. SUV in biopsy confirmed cancer lesions up to 14 min post injection while decreasing in inguinal lymph nodes interpreted as benign. Furthermore, it was very useful in differentiating local recurrences from confounding blood pool and urinary activity.^10^ Although more data need to be obtained, it appears that ^18^F-choline PET/CT is highly efficient in preoperative management regarding N and M staging of prostate cancer once metastatic disease is strongly suspected or documented.^11^ ^18^F-choline PET/CT could be useful in the evaluation of patients with prostate cancer who are at high risk for extracapsular disease, and it could be used to preoperatively exclude distant metastases.^12^

Patients with persistent elevated PSA and repeated negative prostate biopsy, (*i.e*. prostate being biopsied at multiple times), were investigated with ^18^F-choline PET/CT to delineate prostate cancer and guide renewed prostate biopsy. In 25% of patients, ^18^F-choline PET/CT allowed the identification of neoplastic prostatic zones.^13^

The sensitivity, specificity and accuracy of ^18^F-choline PET/CT in the detection of bone metastases in prostate cancer are 74%, 99% and 85%, respectively. ^18^F-choline PET-CT may be superior to bone scintigraphy for the early detection of metastatic bone disease due to the detection of bone marrow metastases.^13^

Out of all patients with carcinoma of the prostate undergoing therapeutic regimes with curative intent, 15–23% will ultimately relapse and 16–35% will need some sort of salvage therapy within 5 years. Of relapsing patients, 50% will have local recurrence and 50% systemic disease with or without local recurrence. Therefore, the localisation of recurrent prostate cancer is critical for selecting a local or systemic therapeutic strategy.^15^ Modern fusion imaging with ^18^F-choline PET/CT has augmented the diagnostic imaging spectrum for the assessment of relapsing prostate cancer. In 60–70% of patients with biochemical relapse, recurrent tumour can be detected and anatomically precisely localised. Imaging with ^18^F-choline PET/CT and MRI possesses a high potential for the early localisation of recurrent prostate carcinoma.^16^

In patients with biochemical relapse after the radical treatment for prostate cancer, ^18^F-choline PET/CT represents a single step, whole-body, non-invasive study that allows disease detection and localisation. Detection sensitivity is probably negatively correlated with serum PSA concentration. Pelosi *et al.* reported that ^18^F-choline PET scan detected the disease relapse in 42.9% of cases (24/56). PET sensitivity was 20% in the PSA ≤ 1 ng/ml subgroup, 44% in PSA > 1 and ≤ 5, and 81.8% in PSA > 5 ng/ml subgroup, respectively.^17^ According to other investigators ^18^F-choline PET/CT is not likely to have a significant impact on the care of prostate cancer patients with biochemical recurrence until PSA increases to above 4 ng/ml. However, in selected patients, ^18^F-choline PET/CT helps to exclude distant metastases when the salvage local treatment is intended.^18^ Most probably doubling time of serum PSA increase is more important as PSA level itself.

^18^F-choline PET/CT seems to be useful also for the evaluation of other cancers with poor FDG uptake, such as hepatocellular carcinoma.^19^

## Conclusions

In future studies some of dilemmas that appear in presented study need to be solved: to correlate PET/CT results with standard prognostic factors and to determine their prognostic significance (correlation of our PET/CT results with starting PSA, clinical T stage, Gleason score in surgically treated/ biopsied patients and PSA doubling time in patients with biochemical recurrence).

^18^F-choline PET/CT seems to be useful imaging modality in patients with prostate carcinoma for demonstrating the spread of the disease preoperatively and to detect local recurrent disease after radical prostatectomy.

## Figures and Tables

**TABLE 1.** Results of ^18^F-choline PET/CT scans in 50 patients with prostate carcinoma

	**Number of patients**	**Prostatic bed (positive)**	**Metastases (positive)**
**After radical prostatectomy**	18	6 (33 %)	2 (10%)
**No surgery**	32	32 (100%)	14 (44%)
**Total**	50	38 (96%)	16 (33%)
